# Effect of Gamma Irradiation on Fat Content, Fatty Acids, Antioxidants and Oxidative Stability of Almonds, and Electron Paramagnetic Resonance (EPR) Study of Treated Nuts

**DOI:** 10.3390/molecules28031439

**Published:** 2023-02-02

**Authors:** Svetlana Momchilova, Adriana Kazakova, Sabina Taneva, Katerina Aleksieva, Ralitsa Mladenova, Yordanka Karakirova, Zhanina Petkova, Mariana Kamenova-Nacheva, Desislava Teneva, Petko Denev

**Affiliations:** 1 Laboratory of Lipid Chemistry, Institute of Organic Chemistry with Centre of Phytochemistry, Bulgarian Academy of Sciences, Acad. Georgi Bonchev Str., bl. 9, 1113 Sofia, Bulgaria; 2 Centre of EPR spectroscopy, Institute of Catalysis, Bulgarian Academy of Sciences, Acad. Georgi Bonchev Str., bl. 11, 1113 Sofia, Bulgaria; 3 Laboratory of Organic Synthesis and Stereochemistry, Institute of Organic Chemistry with Centre of Phytochemistry, Bulgarian Academy of Sciences, Acad. Georgi Bonchev Str., bl. 9, 1113 Sofia, Bulgaria; 4 Laboratory of Biologically Active Substances, Institute of Organic Chemistry with Centre of Phytochemistry, Bulgarian Academy of Sciences, 139 Ruski Blvd., 4000 Plovdiv, Bulgaria

**Keywords:** gamma irradiation, almonds, nut oil, fatty acids, antioxidants, oxidative stability, EPR

## Abstract

Gamma irradiation has been applied as an efficient and inexpensive method for the sterilization of nuts for years. However, along with the benefits of such treatment, negative effects are possible because of the formation of reactive oxygen species with a toxic effect on important biologically active substances. Because of the scarce and contradictory information in the literature about gamma-irradiated almonds, the aim of our work was the examination of the lipid changes, antioxidant activity, and oxidative stability of almonds treated by 10 and 25 kGy gamma rays, as well as changes in intensity of the EPR spectra as an indicator for the stability of radiation-induced free radicals. The results revealed no significant differences in the EPR spectra of almonds treated at 10 and 25 kGy doses, neither in their intensity nor in kinetic behaviour. The EPR signals decayed exponentially over 250 days, with a decreasing of central line by 90%, with satellite lines by about 73%. No significant changes in the fat content, fatty acids composition, and acid value of irradiated almonds were observed. However, the amount of (alpha)tocopherols decreased from 292 to 175 mg/kg, whereas the conjugated dienes and trienes increased, K_232_ from 1.3 to 3 and K_268_ from 0.04 to 0.15, respectively, with the increasing of irradiation dose. The same was observed for total polyphenols in defatted almonds (1374 to 1520 mg/100 g), where in vitro antioxidant activity determined by ORAC and HORAC methods increased from 100 to 156 µmol TE/g and from 61 to 86 µmol GAE/g, respectively. The oxidative stability of oil decreased from 6 to 4 h at 120 °C and from 24.6 to 18.6 h at 100 °C (measured by Rancimat equipment). The kinetic parameters characterizing the oxidative stability of oil from 10 kGy irradiated almonds were studied before and after addition of different concentrations of ascorbyl palmitate as a synergist of tocopherols. Its effectiveness was concentration-dependent, and 0.75 mM ensured the same induction period as that of non-irradiated nut oil. Further enrichment with alpha-tocopherol in equimolar ratio with palmitate did not improve the oil stability. In conclusion, gamma irradiation is an appropriate method for the treatment of almonds without significant changes in fat content and fatty acids composition. The decreasing of oxidative stability after higher irradiation could be prevented by the addition of ascorbyl palmitate.

## 1. Introduction

Nuts are accepted as a superfood because of their high content of healthy nutrients, especially unsaturated fatty acids, phytosterols, antioxidants, dietary fibers, proteins, and microelements [1,2]. Among nuts, almonds (*Amygdalus communis* L.) are one of the favorites because of their excellent taste and increasingly demonstrated health benefits [3,4]. To prevent them from developing pathogenic microorganisms and, thus, to prolong easy and cheap shelf life, almonds can be treated by gamma rays [5,6,7]. Regulations on irradiation conditions and requirements, as well as process control, were introduced by Codex Alimentarius, the International Atomic Energy Agency, the World Health Organization, and the European Comission, and in 2003, the Codex Commission ratified the Codex General Standard for Irradiated Foods [8]. However, along with the benefits of such treatment, negative effects are possible, too, because of formation of reactive oxygen species (ROS) with a strong toxic effect on important biological structures, such as cellular lipid membranes, proteins, etc. [9]. When the generation of ROS is accompanied by the formation of various advanced glycation end products, it weakens the immune system and provokes diseases, such as diabetes, atherosclerosis, neurodegenerative or vascular diseases, chronic renal failure, etc. [10,11]. The normal physiology of the human body has a delicate balance between the production of ROS and their elimination by the protective effects of antioxidants. Although many investigations deal with lipid composition and antioxidant contents, only single papers can be found in the literature concerning the effects of gamma irradiation on almonds; moreover, these studies are rather conflicting. Along with the expected increase in peroxide value [12], one paper announces it decreasing with increasing of the radiation dose [13]. Regarding the fatty acid composition, according to some authors, gamma irradiation up to 10 kGy does not cause any changes [14], but according to others, monoenoic acids increase without any change in dienoic acids [15]. Moreover, there is a publication vice versa, i.e., monoenoic acids decrease, while polyenoic have not been changed [16]. All these contradictions in the available published data require a detailed study of the treated almonds to establish whether gamma irradiation indeed changes their composition. On the other hand, the oxidation processes are strongly affected by antioxidants, and these interactions have been explored extensively for many years. However, to the best of our knowledge, there are no data in the literature about the stabilization of oil from irradiated almonds by addition of individual antioxidants or their mixtures. Thus, the aim of our investigation was to study: (i) the effect of gamma irradiation on the fat content, fatty acids composition, and oxidative stability of oil from gamma-treated almonds, including the testing of antioxidants for oil stabilization after irradiation; (ii) the electron paramagnetic resonance (EPR) spectroscopic study of gamma-treated almonds, regarding the stability of free radicals induced by irradiation, as well as the antioxidant activity of defatted nuts. Two doses of irradiation were chosen for our experiments, namely 10 and 25 kGy, with the first as a maximum permitted level for food gamma treatment [8] and the latter as a much higher dose, allowing for the comparison of results and some assessment of irradiation effects on almonds lipids.

## 2. Results and Discussion

### 2.1. Electron Paramagnetic Resonance (EPR) Study of Gamma-Irradiated Almonds

#### 2.1.1. Characteristics of EPR Spectra

EPR spectroscopy is a method where electromagnetic radiation with microwave frequency is absorbed by the atoms in the molecules or solids having electrons with unpaired spins. Therefore, EPR allows for the examination of free radicals and paramagnetic centers in solid, liquid, or gaseous states, as well as the bi-radicals, atoms, and ions of transition metals. The EPR method possesses high sensitivity and resolution; moreover, it is nondestructive. It can detect paramagnetic particles in concentrations at 10^−11^–10^−12^ M levels and, therefore, has been widely used in physics, chemistry, biology, medicine, and other fields of study. The EPR is based on the well-known effect of Zeeman, where an intense external magnetic field produces a difference between the energy levels of the electron spins m = +½ and m = −½, resulting in resonance absorption of an applied microwave beam in the spectrometer. The difference in the energies of the two spin states, ΔE = gβH, depends on the intensity of the applied magnetic field H and on the spectroscopic splitting factor g. The coefficient β is the Bohr magneton. The g value is an intrinsic characteristic of the paramagnetic center and its local coordination. It is the most important feature because it defines the position of line in the magnetic field. Thus, the g value determines the types of paramagnetic center, free radicals, biradicals, ions of transition and rare earth elements, point defects, etc. EPR spectra are conventionally displayed as the first derivative of the absorption, with respect to the applied magnetic field.

**Figure 1:**
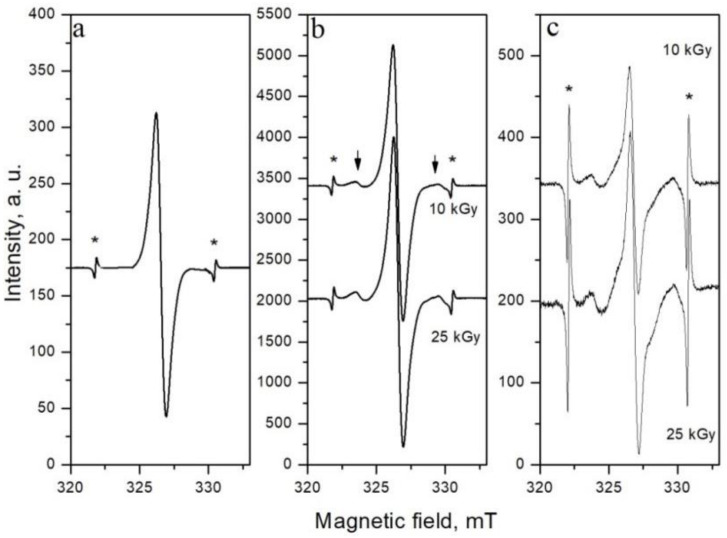
EPR spectra of almonds: (**a**) non-irradiated sample; (**b**) immediately after gamma irradiation; (**c**) 250 days after gamma irradiation; (*) the lines of Mn^2+^ ions.

Non-irradiated shell sample of almonds shows one singlet line characterized with a *g*-factor of 2.0026 (Figure 1a). This line is observed in all non-irradiated foodstuffs of plant origin and is attributed to the free radicals of semi-quinones, produced by oxidation of plant phenolic groups present in polyphenols or lignin [17]. Almond samples irradiated at 10 and 25 kGy exhibited a typical “cellulose-like” EPR spectrum containing an irradiation-specific line pair of the cellulose radical (satellite lines marked with arrows in Figure 1b, assumed to arise from the C(5) carbon-centered cellulose radical [18]), spaced about 6 mT from each other, and increased intensity of the central line. The presence of the characteristic “cellulose–like” EPR spectrum is considered in EU Standard 1787 as unambiguous evidence for the previous radiation treatment of plant foodstuffs [19]. The second radiation induced signal appeared as a strong singlet with *g*-factor of 2.0055 and overlapping with natural weak line and “cellulose-like” EPR spectrum. This admission for a second radiation-induced EPR singlet line was confirmed by the different decay rate constant of the disappearance of the two radiation-induced features [20,21]. In addition, the spectrum of Mn^2+^ ions was observed (marked with asterisks in Figure 1), whose fifth line overlapped with the right satellite line. This manganese spectrum confirmed the presence of a high amount of this mineral in almonds. As can be seen in Figure 1c, 250 days after irradiation, the central line significantly decreased, compared to satellite lines, but the latter was still visible. When the satellite lines disappeared, the manganese spectrum could be used as an internal naturally present standard for the identification of gamma-sterilization [22]. Thus, gamma irradiation did not induce a large difference in the amount of free radicals at 10 and 25 kGy doses, since no significant difference in intensity of EPR spectra was observed. These results could be explained by the saturation of the signal at higher doses or a higher extent of the recombination of the free radicals because of the large number of closer-situated radicals in the 25 kGy samples.

#### 2.1.2. Kinetic Study of EPR Spectra

**Figure 2:**
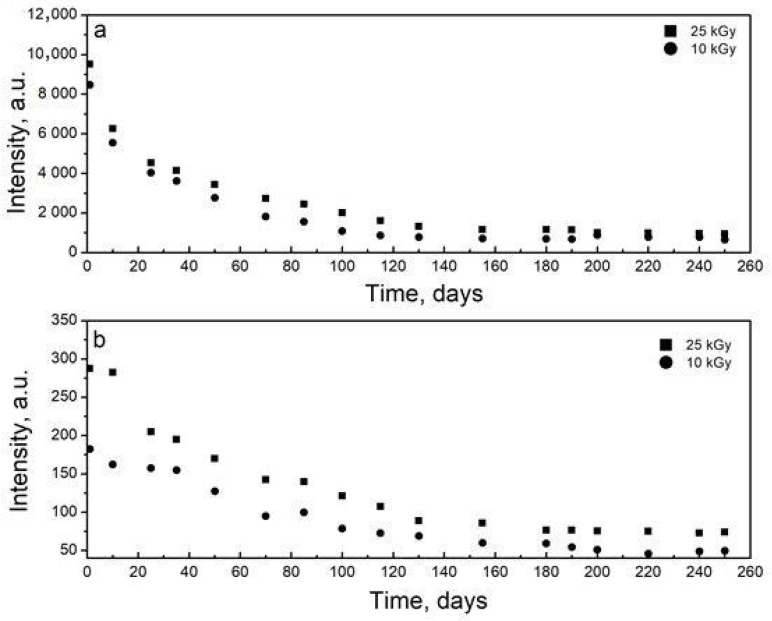
Fading kinetics during 250 days of EPR spectra intensity after gamma irradiation of almonds: (**a**) central line; (**b**) satellite lines.

In order to establish the time stability of the radiation-induced EPR signals, their decay kinetics were studied for a period of 250 days after the irradiation of the almonds. According to the results, the main EPR signals decayed exponentially with time, with a slightly higher intensity in the 25 kGy irradiated samples than that of the 10 kGy dose (Figure 2a). Similarly, the satellite lines were more visible for almonds irradiated with 25 kGy, and this trend persisted until the end of the study (Figure 2b). The kinetic investigation revealed that the central line decreased by about 90%, while the satellite lines decreased by about 73%. The different behavior of central and satellite peaks proved the case for at least two radiation-induced signals. No significant difference in the kinetic behavior for almonds irradiated at 10 and 25 kGy was observed.

#### 2.1.3. Free Radical Scavenging Activity (FRSA) Assay of Defatted Almonds

**Figure 3:**
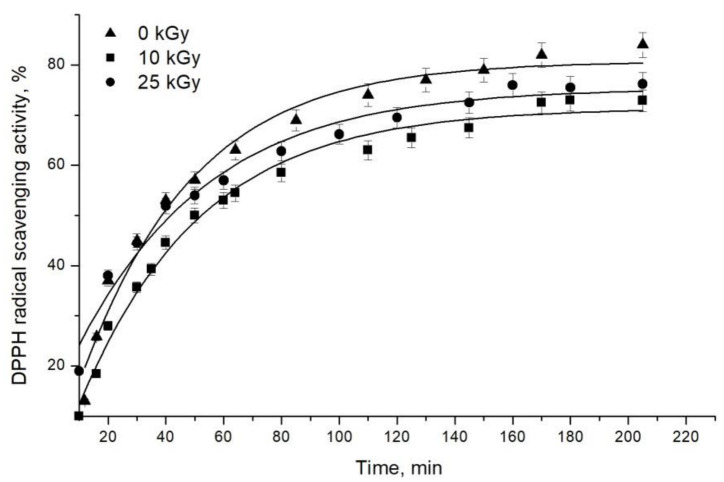
DPPH free radical scavenging activity of defatted almonds before (0 kGy) and after (10 and 25 kGy) gamma irradiation.

The time dependence of 1,1-diphenyl-2-picrylhydrazyl (DPPH) free radical scavenging activity of irradiated (10 and 25 kGy) and non-treated defatted almonds is presented in Figure 3. As can be seen, a gradual increase in the antiradical activity over time was established. Up to about 40 min, the dependence was linear, whereas after approximately 160 min, a plateau was formed. The results show that the irradiation FRSA decreased by 9% and 4% in samples from almonds irradiated by 10 and 25 kGy, respectively. Many authors designate a strong connection between the content of nut phenolics and antioxidant abilities [23,24]. In accordance with our study, Moosavi et al. reported the decreasing of the radical scavenging capacity of almond hull extracts after gamma irradiation with 6 and 10 kGy, probably due to the reduction in tannin content [23]. In contrast, Harrison and Were observed an increase of phenolic content and antioxidant capacity in irradiated almond skin extracts, compared to non-irradiated ones [25].

The generation of free radicals is one of the main effects of gamma irradiation because of the ionizing radiation releases electrons from molecules. Since antioxidant molecules scavenge the gamma-induced radicals in the course of the process, they are exhausted. On the other hand, no changes in the total phenolic content in the 10 kGy sample was detected (see Section 2.3.2); therefore, the exhaustion of antioxidants could not be compensated for, and this resulted in lower antiradical activity, expressed by % DPPH FRSA (Figure 3). However, in 25 kGy sample, an increase of total phenols amount was registered (Section 2.3.2). It could be assumed that it compensated for the loss of activity due to depletion of antioxidants after the higher dose irradiation.

Some studies indicate a number of factors that could be responsible for the observed differences in the results of the irradiation effect on FRSA for nuts. The genotype, geographical and environmental conditions, cultivation technique that occur during the pre-harvest period, post-harvest storage conditions [26], extraction solvent and procedure [27], treatment of the sample (drying, roasting, irradiation) [28], dose of irradiation, etc., may affect the chemical composition of plant food and play decisive roles in the phenolic content. The irradiation might break the covalent bounds in polyphenols, and they probably become decomposed to compounds without antioxidant abilities. This process is more pronounced in 10 kGy irradiated samples.

### 2.2. Effect of Gamma Irradiation on Fat Content, Fatty Acids Composition, and Acid Value

The results for fat content, fatty acids composition, and acid value of oil from irradiated almonds are given in Table 1. As can be seen, there is no change in the fat content of ~50% at the three irradiation doses (0, 10, and 25 kGy). The same has been observed by other authors, as well, for almonds irradiated up to 10 kGy, without data available for 25 kGy [15,29,30].

As for fatty acids, the results yet published are conflicting, since some have reported an increasing of monoenoic acids [15] vs. decreasing by others [16], or no changes [14] up to 10 kGy, without information for 25 kGy. Our investigation established no significant difference in the fatty acids composition of oil from almonds irradiated at doses of 10 and 25 kGy, compared to non-irradiated nuts (Table 1). Similarly, the acid value, of which an increase could be considered an indicator for degradation processes, was not changed, irrespective of increased radiation dose. Our results were in agreement with the data of other authors for almonds irradiated up to 10 kGy [13], although information regarding the increasing of free fatty acids in gamma-treated oil was found, as well [15].

### 2.3. Effect of Gamma Irradiation on Antioxidants and Oxidative Stability of Almond Oil

Lipid oxidation is considered the most important problem affecting edible oils because it engenders not only a loss of their organoleptic characteristics, but also causes the destruction of fatty acids and fat-soluble vitamins and results in the formation of physiologically harmful substances. Even in the middle of the last century, it was established that the level of oxidation at the early stages of the process gave information about the later behavior of the oil [31]. For that reason, we have investigated the oxidative stability of oil from gamma-irradiated almonds by a complex approach, including the main antioxidants (in oil and in the defatted nut cake), the primary products of lipid oxidation (estimated by conjugated dienes and trienes absorbance, and peroxide value) and the kinetics of their accumulation (the Induction period), and the overall oxidative stability determined by secondary and final oxidation products (measured by Rancimat equipment).

#### 2.3.1. Tocopherols in Oil from Irradiated Almonds

Tocopherols are among the most widespread antioxidants in plants, and they typically occur as a mixture of four isomers (alpha-, beta-, gamma-, and delta-tocopherol) in different proportions [32]. In almonds, including the nuts investigated here, the alpha-isomer is predominant, whereas gamma- and delta-tocopherols are in negligible amounts and beta-isomer is usually not detected [33,34]. Our results (Table 1) revealed a significant decreasing of alpha-tocopherol content, from 292 to 175 mg/kg, with an increasing of gamma irradiation dose to 25 kGy, which suggests its high antioxidant activity. The same trend was reported by other authors for almond kernels treated with 8 kGy gamma rays [14,15].

#### 2.3.2. Polyphenols and Antioxidant Activity of Defatted Almonds

According to some publications, gamma irradiation reduces the amount of polyphenols [35]. However, other authors have observed increased phenolic content and antioxidant activity of almond extracts after 4 kGy and 12 kGy gamma-treatment [25]. The irradiated almonds had a lower degree of oxidation, compared to control samples, indicating that the phenolic compounds in almond husks inhibited the oxidation of lipids [25]. In our study (Table 2), a similar trend of increasing from 1374 to 1520 mg/100 g of polyphenols in defatted almonds was detected at dose of 25 kGy. The antioxidant activities measured in vitro by the ORAC and HORAC methods also increased from 100 to 156 µmol TE/g and from 61 to 86 µmol GAE/g, respectively. The increase in antioxidant activity could be attributed either to the increased ability of a plant to produce radioprotective antioxidants or to the increased extractability of phenolic antioxidants from the degraded plant matter [36]. The former could be attributed to increased enzyme activities, which, in the case of almonds, is not very plausible, due to the low water content of dried nuts. Therefore, the increased extractability of phenolic antioxidants from the plant matrix is the most plausible reason for the elevated ORAC and HORAC values [36]. This has already been observed for irradiated almond skins, which reveled higher phenolic content and antioxidant activity, in comparison to untreated samples [25]. Furthermore, the gamma rays could alter the phenolic profile of irradiated samples, causing the cleavage of glycosidic bonds in flavonoids or degradation of tannins. For example, quercetin-3-O-glucoside and rutin decreased dose-dependently in irradiated rose hip fruits, which was accompanied by an increased content of the stronger antioxidant—quercetin [37]. Additionally, gamma irradiation caused the degradation of tannins in soybean samples, which resulted in the release of low-molecular polyphenols with an altered antioxidant activity [35].

#### 2.3.3. Conjugated Dienes and Trienes in Oil from Irradiated Almonds

Conjugated dienes and trienes are primary products of lipid oxidation and, hence, are important indicators for initial stages of oxidation processes. The data in Table 3 show the increasing of their amounts after irradiation, K_232_ from 1.3 to 3 and K_268_ from 0.04 to 1.1, respectively, with the increasing of irradiation dose, in parallel with the decreasing of tocopherol content (Table 1).

#### 2.3.4. Oxidative Stability of Oil from Irradiated Almonds

**Figure 4:**
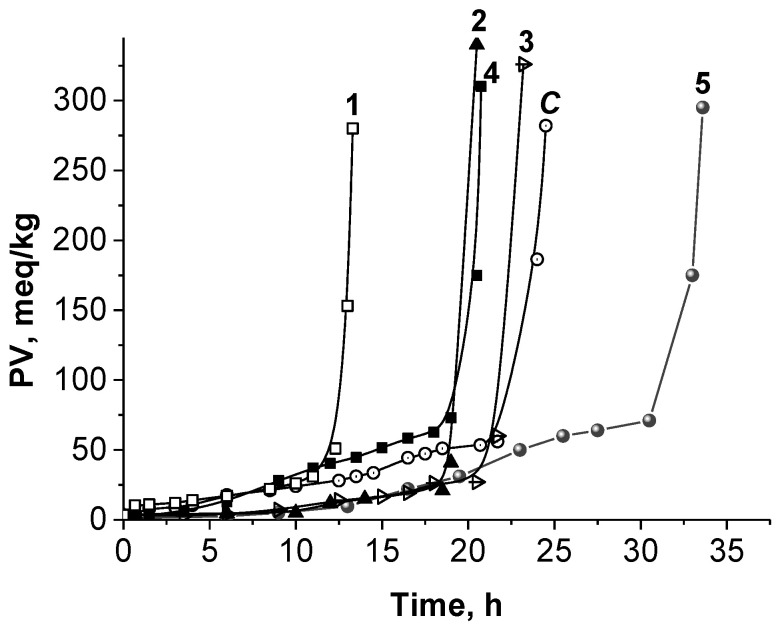
Kinetics of lipid hydroperoxides accumulation during autoxidation at 100 °C of oil from untreated (control, C) and gamma-irradiated (10 kGy) almonds before (curve 1) and after addition of ascorbyl palmitate at 0.5 mM (curve 2) and 0.75 mM (curve 3); addition of equimolar mixture of α-tocopherol and ascorbyl palmitate at 0.5 mM to irradiated (curve 4) and non-irradiated (curve 5) sample.

The oil stability has been recognized as an even more important quality parameter than the current extent of oil oxidation presented by the peroxide value. In our work, the oxidative stability of oil from gamma-treated almonds was evaluated by two manners: (i) expressed as the oxidative stability index (OSI, measured by Rancimat apparatus); (ii) by the induction period (IP) of autoxidation. The OSI is related to the final stages of the oxidation processes, in contrast to the IP, which includes the increasing of the peroxide value (PV) over time. The OSI values of oils from irradiated almonds oxidized at 100 °C and 120 °C are given in Table 3. A significant decrease of oxidative stability, presented by OSI, was observed at both temperatures from about 25 to 19 h and from 6 to 4 h, respectively (Table 3). Regarding the PV, its increasing after almonds irradiation up to 10 kGy has been reported in the literature [16,38]. In addition, our investigations comprised the measurement of PV during the autoxidation of oil from non- and gamma-treated almonds, and accordingly, the determination of the respective IP (Table 4). Moreover, the effect of ascorbyl palmitate added to the oil as a single antioxidant or as a mixture with alpha-tocopherol was studied (Table 4, Figure 4) as a chance for the stabilization of oil. Figure 4 presents the kinetics of lipid peroxides (LOOH) accumulation during the bulk phase autoxidation of oil from almonds irradiated with a dose of 10 kGy in the absence and in the presence of ascorbyl palmitate, AscPH (0.5 mM and 0.75 mM), as well as in equimolar mixture (1:1) with alpha-tocopherol, TOH (AscPH: TOH), at 0.5 mM.

Comparing the control sample (Figure 4, C) with the irradiated one (Figure 4, curve 1), it is seen that latter has reduced the antioxidant potential, most likely due to the involvement of its antioxidants (as commented in Section 2.3.1, Table 1) in the capturing of the free radicals generated by gamma rays.

Kinetic parameters obtained after processing the curves are presented in Table 4. Induction periods (IP), i.e., time in which the concentrations of antioxidants were fully consumed, were determined as a cross point for the tangents to both parts of the kinetic curves. Oxidation rates were found by the tangents to the initial phase, i.e., the linear part. Thus, the non-irradiated sample had an induction period of 22 h (Table 4). Enrichment with antioxidants’ mixture of ascorbyl palmitate and alpha-tocopherol (AscPH: TOH, 1:1) ensured the prolongation of IP to 32 h, i.e., with 10 h. On the other hand, the same antioxidants’ mixture extended the IP of gamma-irradiated sample from 13 h to 20 h, i.e., with 7 h (Table 4). A very similar result (18 h ± 2) for IP was achieved after the addition of 0.5 mM ascorbyl palmitate. However, the enrichment of gamma-treated oil with 0.75 mM ascorbyl palmitate resulted in a significantly improved IP to a value practically the same as that of the non-irradiated control sample. These results reveal the antioxidants’ potential for the stabilization of oil, even after the gamma-treatment of nuts. In this study, just ascorbyl palmitate and alpha-tocoperol in the given concentrations were selected for the experiments, as the permitted and common antioxidants widely used in the food industry.

Regarding the initial oxidation rate, the control and irradiated samples had the same value for R (0.3 × 10^−6^ M/s, Table 4). The addition of ascorbyl palmitate to gamma-treated oil resulted in the decreasing of R, as opposed to its mixture with tocopherol. So, in spite of practically the same induction periods (Figure 4, curves 2 and 4), the 0.5 mM AscPH individually ensured a 2.5-fold lower oxidation rate, compared to that in the mixture with TOH (Table 4). In other words, the additional enrichment with alpha-tocopherol, in this case, did not lead to a better stabilization of oxidizable lipid substrate.

**Figure 5:**
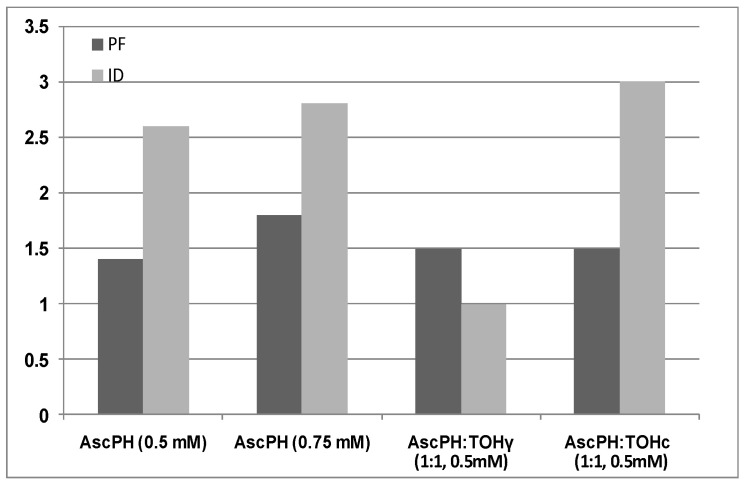
Protection factor (PF) and inhibition degree (ID) of stabilized gamma-irradiated samples with ascorbyl palmitate (AscPH) at 0.5 mM and 0.75 mM, as individuals and in equimolar mixture with alpha-tocopherol (TOH; AscPH: TOH_γ_), compared with PF and ID of the stabilized control sample (AscPH: TOH_C_).

The antioxidants effect on oil stabilization can be seen in Figure 5, too, where the values of the protection factor (PF) and inhibition degree (ID) are graphically presented. The protection factor indicates how many times the antioxidant increases the oxidation stability of lipid sample. The PF is determined as the ratio between the induction periods in the presence (IP_A_) and in the absence of the antioxidant, as follows:

PF_C_ = IP_A_/IP_C_ and PF_γ_ = IP_A_/IP_γ_, where IP_C_ and IP_γ_ are the induction periods of non-irradiated (0 kGy) control and of irradiated (10 kGy) oil samples, respectively. On the other hand, the antioxidant reactivity expresses the possibility of the antioxidant to take part in side reactions of the oxidation process, i.e., to change the initial oxidation rate, and can be presented by the inhibition degree (ID), as follows:

ID_C_ = R_C_/R_A_ and ID_γ_ = R_γ_/R_A_, where R_C_ and R_γ_ are the initial rates of non-irradiated (0 kGy) control and of irradiated (10 kGy) oil samples, respectively. R_A_ is the rate in the presence of the studied compounds at certain concentrations, i.e., 0.5 mM or 0.75 mM AscPH or 0.5 mM AscPH:α-TOH (1:1).

As can be seen in Figure 5, the protection factor for almond oil obtained from irradiated and from non-irradiated nuts in the presence of the studied mixture is practically the same (value of 1.5). However, the inhibition degree of the latter is three-fold higher, which means that the level of side reactions responsible for generation of hydroperoxides (LOOH) is much lower. Further experiments with different concentrations and/or other antioxidants and mixtures might reveal, to a greater extent, their capacity for the stabilization of oil from gamma-irradiated nuts.

## 3. Materials and Methods

### 3.1. Samples and Reagents

Almonds were obtained from a local producer of Nova Zagora region, Bulgaria, and were checked by electron paramagnetic resonance (EPR) spectroscopy (see below) that they had not previously been gamma-irradiated. Until analyses, the shelled nuts were stored in polyethylene bags in dark cool place. All reagents were of analytical grade (Merck, Darmstadt, Germany), and solvents were of HPLC grade (Merck, Sigma-Aldrich). Reference fatty acid methyl esters, alpha- and gamma-tocopherols, ascorbyl palmitate, gallic acid, DPPH, Trolox (6-hydroxy-2,5,7,8-tetramethylchroman-2-carboxylic acid), and other reagents for ORAC and HORAC methods were from Sigma-Aldrich Co. (St. Louis, MO, USA).

### 3.2. Gamma Irradiation of Almonds

Shelled almonds were placed in polyethylene bags (air atmosphere) and were gamma-treated by doses of 10 and 25 kGy at 1.230 kGy/h rate. For the purpose, a radionuclide ^60^Co source (gamma ray energies 1.17 and 1.33 MeV) was used with 8200 Ci activity in a mobile irradiation chamber (13.5 × 22 cm, volume of 4.0 L) of semi-industrial radiation system “NIGU-7”. The gamma ray doses were confirmed by alanine dosimeters (Kodak BioMax), which were measured by an EPR spectrometer, E-scan Bruker, and calibrated in units of absorbed dose in water. Three dosimeters were placed at each point. The irradiation was carried out at the National Center for Radiobiology and Radiation Protection, Ministry of Health (Sofia, Bulgaria). Almonds were subjected to analyses immediately after their irradiation. Portions of non-irradiated nuts were used as control samples.

### 3.3. Electron Paramagnetic Resonance (EPR) Investigations of Almonds

#### 3.3.1. EPR Measurements

Almond shells were used after cutting them into small pieces and then placed in quartz EPR tube. For the kinetic experiments, the samples prepared immediately after irradiation were stored, and their spectra were recorded at defined times: every day in the first 10 days, and then once every 10 days. The EPR spectra were recorded as a first derivative of the absorption signal of JEOL JES-FA 100 EPR spectrometer at room temperature. The spectrometer operated in X–band equipped with a standard TE_011_ cylindrical resonator. The EPR spectra were recorded at modulation frequency of 100 kHz, microwave power 0.4 mW, modulation amplitude 0.4 mT, sweep 15 mT, time constant 0.3 s, and sweep time 2 min.

#### 3.3.2. Determination of Antiradical Activity (DPPH Free Radical Scavenging Activity)

##### Preparation of Extracts

Totals of 7.5 mL absolutely pure ethanol and 2.5 mL distilled water were added to 0.5 g dry residue of defatted almonds (non-irradiated and irradiated with 10 kGy and 25 kGy, respectively). These samples were incubated for 24 h at room temperature without air access and then were filtered before further investigations. Freshly prepared extracts were used for each experiment.

##### Measurements

Totals of 1 mL almond extract and 1 mL 0.002 M ethanolic solution of DPPH were mixed and transferred to a capillary tube in a definite time interval. The capillary tube was sealed and placed inside a standard EPR quartz tube in the EPR cavity. The control sample contained 1 mL ethanolic solution of DPPH and the same amount of ethanol, instead of extract. The changes in EPR spectrum intensity were monitored over a period of 4 h. The percent of the DPPH radicals scavenged by nut extracts was calculated according to the equation:

$$\mathrm{Scavenged}\;\mathrm{DPPH}\;\mathrm{radicals}\;\left(\%\right)=\left[\frac{I_{0}-I}{I_{0}}\right]\times 100$$

where I_0_ was the intensity of the second peak of DPPH signal of the control sample and I was the intensity of the second peak of the same EPR spectrum after addition of the tested substance.

### 3.4. Chemical Characterization of Almonds

#### 3.4.1. Extraction of Oil and Determination of Fat Content

Portions of about 30 g (precisely weighted) unshelled almonds were ground and extracted in Soxhlet apparatus for 8 h with *n*-hexane [39]. The solvent was distilled in rotary evaporator, and the residue was weighted to calculate the fat content by the equation:

$$\mathrm{Fat}\;\%=\left(\frac{m_{\mathrm{oil}}}{m_{\mathrm{nuts}}}\right)\times 100$$

where m was the mass (g) of the residue (oil) and the initial sample (nuts), respectively. The oil samples were kept in −20 °C until analyses.

#### 3.4.2. Determination of Fatty Acids Composition

Fatty acids composition was determined by gas chromatography (GC) with a flame ionization detector after acid-catalyzed transesterification of oil to methyl esters [40]. Prior to GC analysis, fatty acid methyl esters (FAME) were purified by preparative thin-layer chromatography on a silica gel plate with a mobile phase of hexane-acetone (100:6 *v*/*v*). GC was conducted on a Shimadzu GC 2030 chromatograph equipped with a flame ionization detector and simplicity wax capillary column (30 m × 0.32 mm × 0.25 μm, Supelco) operating from 170 °C to 260 °C at 2 °C/min and 5 min and held at final temperature. The injector and detector temperatures were 260 °C and 280 °C, respectively. Nitrogen was used as a carrier gas at a flow rate of 0.6 mL/min, split 1:50. The peaks identification was according to retention times, compared to that of a standard mixture of FAME.

#### 3.4.3. Determination of Tocopherols

Tocopherols were measured directly by high-performance liquid chromatopgraphy (HPLC) of hexane solution of oil (400 µg sample size). The analyses were performed by Agilent 1100 apparatus equipped with Nucleosil 100-5 (250 mm × 4.6 mm, 5 µm) column, connected with a pre-column EC 4/3 Nucleosil 100-5 (Macherey-Nagel) and DAD detector at 292 and 298 nm for *α*- and *γ*-isomers, respectively. The mobile phase was a mixture of hexane and tetrahydrofuran (96:4 *v*/*v*) at flow rate of 1 mL/min [41]. Quantitation was performed using a calibration curve of tocopherol in a range from 0.025 to 0.05 mg/mL, and the results were presented as (mg tocopherol/kg oil).

#### 3.4.4. Determination of Total Polyphenols and Antioxidant Activity by ORAC and HORAC Methods

The content of total polyphenols was determined according to the method of Singleton and Rossi with Folin–Ciocalteu’s reagent [42], using calibration curves of gallic acid in the range from 10 to 200 μg/mL. Oxygen radical absorbance capacity (ORAC) and hydroxyl radical averting capacity (HORAC) were measured according to the methodology used by Denev et al. [43]. Results were presented as micromole Trolox equivalents (µmol TE) and micromole gallic acid equivalents (µmol GAE) per gram sample for ORAC and HORAC, respectively. The measurements were carried out on a FLUOstar OPTIMA plate reader (BMG Labtech, Germany).

#### 3.4.5. Determination of Conjugated Dienes and Trienes Content

The content of conjugated dienes and trienes (K_232_ and K_268_) was evaluated by their absorbance at 232 nm and 268 nm, respectively, of 1% solutions of oils in *iso*-octane using Cecil Series 8000 UV/VIS spectrophotometer with reference to pure solvent [44]. The results were presented as K_232_ and K_268_, respectively.

#### 3.4.6. Determination of Acid Value, Peroxide Value, Induction Period and Oxidative Stability Index

The acid value (AV) was estimated by titration of oil with ethanolic KOH [45]. Peroxide value (PV), expressed as meq O_2_/kg oil, was determined according to the modified iodometric method of Yanishlieva et al. [46]. The induction period (IP) was evaluated by the following procedure: 2 g oil were oxidized in a glass vessel at 100 °C by blowing a stream of air and aliquots of oil were taken at regular intervals for estimation of peroxide value. Increasing of PV during time was monitored graphically. The induction period (expressed by hours) was fixed by the tangent method of Le Tutour and Guedon [47]. On the other hand, oxidative stability index (OSI) was determined by Rancimat equipment (Metrohm, Switzerland), with 20 L/h air flow at temperatures of 100 °C and 120 °C.

### 3.5. Statistical Analysis

Measurements were carried out in triplicate. Results are presented as a mean value of 3 parallel determinations ± standard deviation and have been compared by Students’ *t*-test (MS Excel 2010 software).

## 4. Conclusions

Gamma irradiation is a simple, efficient, and safe method for the sterilization of nuts. It has been found that the radiation treatment of almonds at doses of 10 and 25 kGy does not cause significant changes in their fat content, fatty acids composition, and acid value. However, increasing the applied dose gamma rays decreases the tocopherols content and, hence, the oxidative stability of oil from irradiated nuts. It has been established that stabilization of this oil can be achieved by the addition of anti-oxidants. Adding 0.75 mM ascorbyl palmitate ensures a longer induction period of oil from irradiated almonds equal to that of non-irradiated nuts. Gamma irradiation slightly affects the antioxidant activity of defatted almonds. Irradiation with 10 kGy has no effect on the polyphenolic content, whereas antiradical activity (expressed as % DPPH) slightly decreases. In samples irradiated with 25 kGy, a slight increase in total phenolic content and the values of ORAC and HORAC has been observed, whereas the impact on DPPH free radical scavenging activity is insignificant. The obtained results by EPR analysis show that EPR spectroscopy can even be used for the identification of the radiation treatment of almonds eight months after irradiation. This is especially important when the time period between the irradiation and the measurement is unknown.

## Figures and Tables

**Table 1:** Fat content, fatty acids composition, acid value, and tocopherols of oil from non-irradiated, 10 kG, and 25 kGy gamma-irradiated almonds.

Almond Oil	Non-Irradiated	10 kGy	25 kGy
Fat content, *w*/*w*%	50.2 ± 0.9 *	50.8 ± 0.5	51.4 ± 0.8
Fatty acids, rel.%			
Palmitic acid (16:0)	5.9 ± 0.1	6.1 ± 0.1	6.1 ± 0.1
Palmitoleic acid (16:1)	0.5 ± 0.1	0.5 ± 0.1	0.5 ± 0.1
Stearic acid (18:0)	1.7 ± 0.1	1.6 ± 0.1	1.8 ± 0.1
Oleic acid (18:1)	64.9 ± 1.0	65.6 ± 1.2	64.0 ± 0.5
Linoleic acid (18:2)	26.9 ± 0.8	26.1 ± 0.7	27.5 ± 1.1
Acid value (mg KOH/g)	1.0 ± 0.1	0.9 ± 0.1	1.1 ± 0.2
*α*-Tocopherol (mg/kg)	292 ± 23 ^a^	215 ± 12 ^b^	175 ± 19 ^c^

* mean value of 3 measurements ± standard deviation. No statistically significant differences between values within each row with fatty acid and acid value were detected (*p* < 0.05). Different letters within tocopherols row indicate statistically significant difference (*p* < 0.05).

**Table 2:** Total polyphenols and in vitro antioxidant activity of non-irradiated, 10 kGy-, and 25 kGy-irradiated defatted almonds.

Defatted Almonds	Non-Irradiated	10 kGy	25 kGy
Total polyphenols (mg/100 g)	1374 ± 35 ^a,^*	1379 ± 36 ^a^	1520 ± 8 ^b^
ORAC (µmol TE/g)	100 ± 3 ^a^	100 ± 5 ^a^	156 ± 3 ^b^
HORAC (µmol GAE/g)	61 ± 3 ^a^	65 ± 2 ^a^	86 ± 6 ^b^

* mean value of 3 measurements ± standard deviation. Different letters within each row indicate statistically significant differences between values (*p* < 0.05). ORAC—Oxygen radical absorbance capacity; TE—trolox equivalents; HORAC—Hydroxyl radical averting capacity; GAE—gallic acid equivalents.

**Table 3:** Conjudated dienes and trienes and oxidative stability index of oils from non-irradiated, 10 kGy, and 25 kGy gamma-irradiated almonds.

Almond Oil	Non-Irradiated	10 kGy	25 kGy
conj. Dienes (A_232_, 1%)	1.3 ± 0.1 *^,a^	2.4 ± 0.1 ^b^	3.0 ± 0.01 ^c^
conj. Trienes (A_268_, 1%)	0.04 ± 0.01 ^a^	0.09 ± 0.01 ^b^	1.1 ± 0.01 ^c^
OSI at 100 °C (h)	24.6 ± 0.2 ^a^	19.5 ± 0.1 ^b^	18.6 ± 0.1 ^c^
OSI at 120 °C (h)	6.0 ± 0.1 ^a^	4.6 ± 0.1 ^b^	4.1 ± 0.1 ^c^

* mean value of 3 measurements ± standard deviation. Different letters within each row indicate statistically significant differences between values (*p* < 0.05). OSI—oxidative stability index measured by Rancimat.

**Table 4:** Kinetic parameters (induction period, IP, and initial oxidation rate, R) characterizing autoxidation at 100 °C of almond oil from irradiated (10 kGy) nuts before and after addition of antioxidants.

Sample	IP, h	R, 10^−6^ M/s	Effect	Curve in Figure 4
control sample **C** (0 kGy)	22 ± 2.0	0.30 ± 0.03	-	C
+0.5 mM AscPH *: TOH (1:1)	32 ± 3.0	0.10 ± 0.02	strong	5
Irradiated sample (10 kGy)	13 ± 1.5	0.31 ± 0.03	-	1
+0.5 mM AscPH	18 ± 2.0	0.12 ± 0.02	strong	2
+0.75 mM AscPH	23 ± 2.0	0.11 ± 0.02	the strongest	3
+0.5 mM AscPH: TOH (1:1)	20 ± 2.0	0.32 ± 0.03	strong	4

* AscPH—ascorbyl palmitate, TOH—alpha-tocopherol.

## Data Availability

Not applicable.
